# Neurological disorders in HIV: Hope despite challenges

**DOI:** 10.1002/iid3.591

**Published:** 2022-02-10

**Authors:** Olivier Uwishema, Georges Ayoub, Rawa Badri, Helen Onyeaka, Christin Berjaoui, Ece Karabulut, Heeba Anis, Christophe Sammour, Fatima E. A. Mohammed Yagoub, Elie Chalhoub

**Affiliations:** ^1^ Oli Health Magazine Organization, Research, and Education Kigali Rwanda; ^2^ Clinton Global Initiative University New York New York USA; ^3^ Faculty of Medicine Karadeniz Technical University Trabzon Turkey; ^4^ Faculty of Medicine Saint Joseph University of Beirut Beirut Lebanon; ^5^ Mycetoma Research Centre Khartoum Sudan; ^6^ Faculty of Medicine University of Khartoum Sudan; ^7^ School of Chemical Engineering University of Birmingham Birmingham UK; ^8^ Faculty of Medicine and Surgery Beirut Arab University Beirut Lebanon; ^9^ Deccan College of Medical Sciences Hyderabad Telangana India; ^10^ Medtech Innovator Riga Technical University Riga Latvia; ^11^ Faculté de Médecine Université Libre de Bruxelles Bruxelles Belgium; ^12^ Faculty of Medicine University of Khartoum Khartoum Sudan

**Keywords:** AIDS, HIV, neurology

## Abstract

**Introduction:**

Human Immunodeficiency virus (HIV) is a virus that causes several diseases by attacking the human immune system. It is transmitted by contact with certain bodily fluids of an infected person, most commonly during unprotected sex, through sharing needles, or from mother to baby during pregnancy, birth or breastfeeding. The central nervous system is not spared from this virus, as HIV has been shown to induce several neurological disorders. However most neurological pathologies (such as dementia, infections, meningitis, and neuropathy) rarely show until late stages, in this case, after the patients develop acquired immunodeficiency syndrome (AIDS). This article aims to review the neurological disorders in the HIV population and the attempts initiated to limit the disease.

**Methodology:**

Data were collected from medical journals published on PubMed, Ovid MEDLINE, Science Direct and Embase bibliographical databases with a predefined search strategy. All articles considering neurological disorders associated with HIV were considered.

**Results:**

To date, the pathogenesis of HIV‐associated neurological complications remains poorly elucidated; thus, imposing a hindrance and limitations on the treatment options. Nevertheless, some studies have reported alterations in dendritic spine as the causative agent for developing brain damage.

**Conclusion:**

HIV remains one of the most serious global health challenges, with neurological manifestations imposing a major concern among patients with HIV. Despite the availability and efficacy of antiretroviral therapies, yet, the risk of developing neurological complications remains relatively high among patients with HIV. Thus, the 2030 HIV vision must focus on further preventive measures to protect HIV patients from developing such neurological complications.

## 1 INTRODUCTION

The human immunodeficiency virus (HIV) infects the body and weakens its immune system. One major characteristic of this virus is to attack the CD4^+^ T lymphocytes and cells from the monocyte/macrophage lineage that leads to the acquired immunodeficiency syndrome (AIDS) if the virus is not appropriately treated.^1^, ^2^ Unprotected sex with someone who has HIV is the most common way to spread the virus, but it can also be transmitted through blood contact and from a mother to her baby.^1^

As HIV and AIDS affect the immune system, the central nervous system (CNS) will suffer as well. Both HIV and AIDS induce various neurological problems, especially when HIV progresses and becomes AIDS.^1^, ^2^

It seems that HIV produces considerable inflammation in the body, damaging the brain and spinal cord, thus preventing neurons from functioning correctly. Added to this, neurological problems normally do not appear until HIV has progressed to the point where a person has AIDS. Demographically speaking, approximately 50% of people with AIDS have HIV‐related neurological problems. Moreover, HIV can cause different conditions such as dementia, infections (meningitis), neuropathy, lymphomas, and psychological diseases.^3^

Antiretroviral treatment can prevent many of the most serious neurological disorders. Interestingly, individuals who get this therapy have less severe neurological and cognitive problems.^4^ The neurological consequences of HIV infection generate significant morbidity and are frequently linked with a high death rate.^4^ This article's objective is to shed light on the different neurological disorders of HIV patients and the endeavors taken to restrain the effects.

## 2 SEVERE NEUROLOGICAL COMPLICATIONS IN PEOPLE LIVING WITH HIV

Despite the advent of advanced antiretroviral therapy, HIV is still notorious for causing neurological complications in AIDS patients as it is neuroinvasive, neurotropic, and neurovirulent. The epidemiological triad consisting of various characteristics of HIV, genetic traits of the host, as well as interactions with the environment, including treatment modalities, determine the course of development of identifiable neurological syndrome in an HIV infected person. Moreover, people living with HIV are at high risk of chronic inflammation, even if they are treated. In fact, their immune cells are continuously stimulated and the primo‐infection induces a sustained lesion of the gut mucosa, resulting in a subtle chronic inflammatory response.^5^ Consequently, as different studies have shown, this chronic condition dysregulates the CNS cells, leading to a loss of function.^6^

Classification of AIDS‐associated neurological syndrome includes primary HIV neurological disease (HIV is the major etiological agent), secondary or opportunistic neurological disease (in which HIV develops into AIDS or interacts with the immune system, resulting in opportunistic infections and tumors), and treatment‐related neurological disease (such as immune reconstitution inflammatory syndrome [IRIS]).^7^ The disease presentation can vary from encephalitis, myelitis, neuropathies with demyelination, aseptic meningitis, severe meningitis due to Toxoplasma, Cryptococcus in the form of opportunistic infections, primary CNS lymphomas, CNS TB (tuberculomas) to visual problems.

The umbrella term HIV‐associated neurocognitive disorder (HAND) is used to describe HIV patients with varying levels of neurocognitive deficits. HAND is further divided into asymptomatic neurocognitive impairment—subclinical decline in cognition, minor neurocognitive disorder, and HIV‐associated dementia—most severe form. These cognitive syndromes have been associated with low antiretroviral adherence and thus represent a significant risk factor for decreased survival.^8^

## 3 DIAGNOSIS AND TREATMENT OF HIV‐RELATED NEUROLOGICAL COMPLICATIONS

Aseptic meningitis is diagnosed by elevated cerebrospinal fluid (CSF) protein with predominantly lymphocytic pleocytosis. Management is symptomatic, antibiotics are used in bacterial coinfections, and acyclovir is used for HIV‐1 or HIV‐2. HIV neurocognitive disorders' diagnosis is mainly clinical. Symptomatic treatment and highly active antiretroviral therapy (HAART) are the standard treatment. Magnetic resonance imaging (MRI) in vacuolar myelopathy (VM) shows severe changes at the thoracic level and cord atrophy in the chronic stage. There is no treatment for VM. MRI is used to diagnose primary CNS lymphoma, cytomegalovirus (CMV) and tuberculosis (TB) spinal cord diseases, Herpes simplex myelitis, human T‐lymphotropic virus 1 (HTLV‐1) associated myelopathy, and progressive multifocal leukoencephalopathy (PML). A biopsy is used to differentiate lymphoma from toxoplasmosis and PML. Cryptococcal meningitis is diagnosed by CSF culture. CMV and TB spinal cord diseases respond well to standard treatment. Herpes simplex myelitis is managed by intravenous acyclovir. HTLV‐1‐associated myelopathy is treated by intravenous corticosteroids. *Toxoplasma gondii* (T.G) myelitis is detected by serology. Sulfadiazine and pyrimethamine are the main drugs in T.G myelitis management. Primary lymphoma may respond to chemotherapy. Cryptococcal meningitis is treated by liposomal amphotericin and 5‐flucytosine, followed by fluconazole. Didanosine, zalcitabine, and stavudine are the most important antiretroviral agents causing peripheral neuropathy. Withdrawal of all three drugs resolves the complications.^9^, ^10^, ^11^, ^12^

## 4 EFFORTS, CHALLENGES, AND RESEARCH BEING TAKEN TO OVERCOME OBSTACLES

Traditional microbiological testing for CNS infections in HIV patients can be challenging, compromising treatment, and outcome.^13^ HIV‐1 infections of the CNS are frequently associated with neurological disorders and cognitive impairment, known as HAND. Even when HIV infection is well‐controlled, neuronal processes in specific brain regions may be deregulated, paving the path for underlying cognitive impairment in combination antiretroviral therapy (cART) treated individuals.

However, it is unknown how HIV infection in a controlled setting causes subtle and localized brain damage. Several studies have found dendritic spine alterations in HIV‐transgenic mice with HAND. This animal model closely resembles human circumstances, such as HIV‐infected humans on cART, in which viral protein production persists despite virus suppression. Even though opioid receptors are present throughout the brain, they may aggravate HAND‐associated synaptodendritic degeneration. In primary neuronal cultures and mu‐opioid receptor mutant animals, morphine exacerbated Tat's excitotoxic effects, including altered calcium and iron homeostasis, mitochondrial instability, and synaptodendritic damage.^14^ Classical opportunistic infections may have different morphologies as a result of immune reconstitution and cART.^15^ IRIS is a common consequence of antiretroviral therapy, particularly in immunocompromised individuals at the start of cART.

What is a metagenomic next‐generation sequencing (mNGS), and how does it work?

Traditional molecular procedures are replaced by sequencing DNA or RNA in a sample. The mNGS may contribute to diagnosing the pathology in HIV patients with atypical clinical and radiographic patterns. Given the wealth of genetic data available on both hosts and pathogens, mNGS offers the potential to improve diagnosis and better understand disease pathophysiology. Some of the problems are high costs to deploy and test samples, a lack of current standardization of methods and data analysis, methodology to establish clinical relevance, and explanatory guidelines for physicians.^13^

## 5 RECOMMENDATIONS

From the public health perspective, a high level of clinical suspicion is recommended to establish an early HIV diagnosis in the setting of an ill‐defined febrile illness and with aseptic meningitis.

Although restoring immunity with ART is the most effective approach to prevent opportunistic neurological infections in HIV‐infected patients, antimicrobial agents should also be administered in immunocompromised individuals. As such, preventing Toxoplasma reactivation is recommended by administering trimethoprim‐sulfamethoxazole. As for the cryptococcal disease, preemptive therapy in positive patients may be appropriate to prevent complications. Furthermore, the risk of invasive meningococcal disease could be significantly decreased with the meningococcal conjugate vaccine.^16^ However, antimicrobial administration could be postponed in a newly diagnosed patient with a highly disturbed psychological state to encourage initial ART therapy adherence.

Regarding neuropsychiatric impairment, supportive psychotherapy and treatment of specific HIV‐associated conditions such as dementia are critical to improving the overall HIV‐related outcome.^17^ Although screening patients for neurocognitive impairment is controversial, establishing a baseline neurocognitive assessment is beneficial in case of subsequent deterioration.^2^, ^18^ Moreover, new biomarkers of neurocognitive impairment are worthy of investigation in future research, along with efforts to optimize HIV therapy within the CNS.^13^ Furthermore, established protocols and extensive research are essential to understand the disease mechanisms, to evaluate the risks of developing complications, and more importantly to choose an adequate treatment. This objective can be achieved by using recent technologies and tools that give a better understanding of the diseases.^19^

## 6 CONCLUSION

HIV remains one of the major and serious health challenges globally, with its increased incidence of infection and lack of suitable treatment options, not to mention the absence of an effective vaccine to prevent its complications. In addition, neurological manifestations, such as neuropathies, infections, and tumors are of major concern among patients with HIV as they increase the deterioration of their physical and mental conditions and lead to higher risks of morbidity and mortality.

Antiretroviral therapy remains the treatment option for these patients as it has been shown to improve neurological manifestations. However, people living with HIV, even if treated, still carry more risks of developing neurological pathologies. Therefore, the 2030 HIV vision should not be limited to getting undetectable viral levels in 90% of patients, but also to have a tool of preventing considerably neurological complications.

## CONFLICT OF INTERESTS

The authors declare no conflict of interest.

## AUTHOR CONTRIBUTIONS

*Conceptualization*: Olivier Uwishema. *Project administration*: Olivier Uwishema. *Data curation*: Olivier Uwishema and Elie Chalhoub. *Formal analysis*: Olivier Uwishema, Elie Chalhoub, Helen Onyeaka, and Rawa Badri. Desining: Olivier Uwishema. *Writing – original draft preparation*: Olivier Uwishema, Georges Ayoub, Rawa Badri, Helen Onyeaka, Christin Berjaoui, Ece Karabulut, Heeba Anis, Christophe Sammour, Fatima E. A. Mohammed Yagoub, and Elie Chalhoub. *Methodology*: Olivier Uwishema. *Writing – review and editing*: Helen Onyeaka and Rawa Badri. All authors have read and approved the final manuscript.
